# Ambient noise levels of seismic stations located in urban agglomerations in central Inner Mongolia, China

**DOI:** 10.1371/journal.pone.0315004

**Published:** 2024-12-05

**Authors:** Wenchao Bao, Quan An, Ye Guo, Lujun Wang, Jianxin Gao

**Affiliations:** 1 Inner Mongolia Autonomous Region Earthquake Administration, Hohhot, Inner Mongolia Autonomous Region, China; 2 Xilinhot Earthquake Monitoring Center Station, Xilinhot, Inner Mongolia Autonomous Region, China; Khalifa University of Science and Technology, UNITED ARAB EMIRATES

## Abstract

Analysis of the continuous ambient noise data collected by a dense network of broadband seismic stations reveals the characteristics of ambient noise in densely populated urban areas. A study conducted in central Inner Mongolia utilized ten broadband stations to investigate two distinct repetitive and intense noise signals with predominant frequencies ranging from 1–20 Hz and 0.01–1 Hz. The ambient noise within the 0.01–20 Hz frequency range was assessed using Probability Density Function (PDF) and Power Spectral Density (PSD) approaches, and the stations were categorized according to their noise levels. The research results indicate that stations located in urban agglomerations are subject to varying degrees of noise interference, with the main sources of interference being human activities, traffic vibrations, and industrial noise. The impact of high-frequency noise on stations is inversely correlated with the distance from the noise source. Among them, four stations are affected by three noise sources. Three stations are affected by two noise sources, and three stations are affected by one noise source. Therefore, the development of urban agglomerations has brought a large number of noise sources to the stations, which greatly affects the data quality of the stations. This finding urges further investigation on the human activities, traffic vibrations, and industrial noise, and suggests that the station construction should be far away from the urban agglomeration.

## 1. Introduction

Seismic stations detect ground vibrations produced by various overlapping sources. The definition of seismic interference varies depending on the focus of individual studies. Majority of seismic networks aim to identify seismic occurrences like earthquakes, volcanic eruptions, quarry explosions, and nuclear blasts, categorizing all other vibrations as background noise [1–3]. Conversely, background noise itself has been the subject of distinct research efforts [4–6]. Noise types can further be classified by their origin, including recording devices, temperature fluctuations, oceanic waves, gravitational variations, wind disturbances, and human actions [3, 7, 8].

The quality of the recorded waveforms is impacted by the amount of background noise, which in turn influences the detection of seismic events. In order to effectively monitor seismic activity, seismic networks must have an understanding of the noise present within the network [3]. Power Spectral Density (PSD) analysis is utilized to calculate the frequency content of the background noise at a particular station. Several models have been developed to analyze noise levels. Peterson’s model is commonly utilized to establish the lower (new low-noise model, NLNM) and upper (new high-noise model, NHNM) boundaries of the recorded noise as a reference point [9]. This model was developed using data from a global database of seismic stations to encompass a wide range of noise levels. McNamara and Buland proposed the Probability Density Function (PDF) method for seismic noise power spectrum, which can be used for measuring station noise levels and waveform quality [10]. It has been applied in daily instrument work detection in stations such as Global Seismic Network (GSN) and Advanced National Seismic System (ANSS). At present, the PDF method is used by the USGS National Earthquake Information Center, the IRIS Data Management Center, and the New Zealand Seismic Network for evaluating the background noise level of seismic stations. It has also been used for analyzing the seismic environmental noise characteristics in the United States, Italy, New Zealand, and Central London [10–13].

Increasing evidence suggests that the seismic noise present in urban areas may be sufficiently strong to corrupt seismic databases utilized for earthquake and tremor detection [14, 15]. Enhancing our comprehension of both natural and human-generated noise is vital for enhancing the identification of minor tectonic events in densely populated regions. Particularly in cities with minimal seismic movements, background noise has emerged as a critical component in contemporary seismology that employs seismic investigations to study subsurface structures and seismic microzonation [16, 17]. Urban ambient noise is influenced by a multitude of human activities such as traffic, public transportation, air traffic, and industrial operations [18, 19]. A comprehensive grasp of the spatial and temporal changes in ground movements induced by urban activities is essential for the effective utilization of seismic noise.

Seismic noises produced by natural or cultural sources exhibit varying frequency content and spatiotemporal features. They can be categorized into two groups: (1) microtremors (frequencies higher than 1 Hz), which are mainly caused by human activities and show consistent daily and weekly patterns [13, 20, 21], and (2) microseisms (frequencies below 1 Hz), primarily triggered by oceanic gravity waves, divided into primary (0.02–0.1 Hz) and secondary (0.1–0.5 Hz) microseisms [22]. Urban seismology has gained significance due to the high levels of ambient noise prevalent in urban regions, leading to an increase in seismic arrays in cities.

This study focuses on the analysis of background noise within the Seismic Network (SN) using data collected from ten continuous stations located in the urban agglomerations of Hohhot-Baotou-Erdos-Ulanqab (HBEU). Our analysis emphasizes short durations (1–20 Hz) as they contain crucial information relevant for civil defense purposes. The ambient noise within the 0.01–20 Hz frequency range was assessed using PDF and PSD approaches, and the stations were categorized according to their noise levels. The primary goal of this research is to examine the rhythmic patterns of ground vibrations in HBEU, exploring the spatiotemporal features and potential sources of ambient noise in this urban agglomeration, and providing scientific recommendations for earthquake management units to renovate earthquake stations.

## 2. Data and methods

### 2.1 Study area

The research area under investigation is situated in northern China, located between the Ordos block and Amurian Block according to Li et al. [23]. Within this region, two primary sectors can be identified: the initial sector encompasses a portion of the northeast of the Ordos block, while the following sector includes a segment of the Hohhot-Baotou basin (faulted basin). The research region primarily lies within the Ordos Basin, a typical cratonic basin formed during the Paleozoic era. The interior of the basin mainly consists of sedimentary rocks from the Paleozoic, Mesozoic, and Cenozoic eras, including sandstones, shales, limestones, etc.

The tectonic framework of the research area is mainly influenced by the North China Platform. During the Paleozoic era, the region experienced multiple tectonic movements that resulted in a series of folds and faults. In the Mesozoic, due to the subduction of the Pacific Plate and the collision of the Indian Plate, the region was subjected to intense compression, leading to the formation of strike-slip faults and thrust faults. In the Cenozoic era, tectonic activity in the region has been relatively weak, mainly characterized by sedimentation within the basin and uplift in the surrounding mountainous areas.

The seismic activity in the research region is relatively high, with earthquakes mainly concentrated along the edges of the basin in the mountainous areas. These earthquakes are primarily related to the region’s tectonic activities, such as the movement of fault zones and adjustments of crustal stress. The risk of earthquake disasters cannot be ignored due to the high population density and urbanization level.

**Fig 1:**
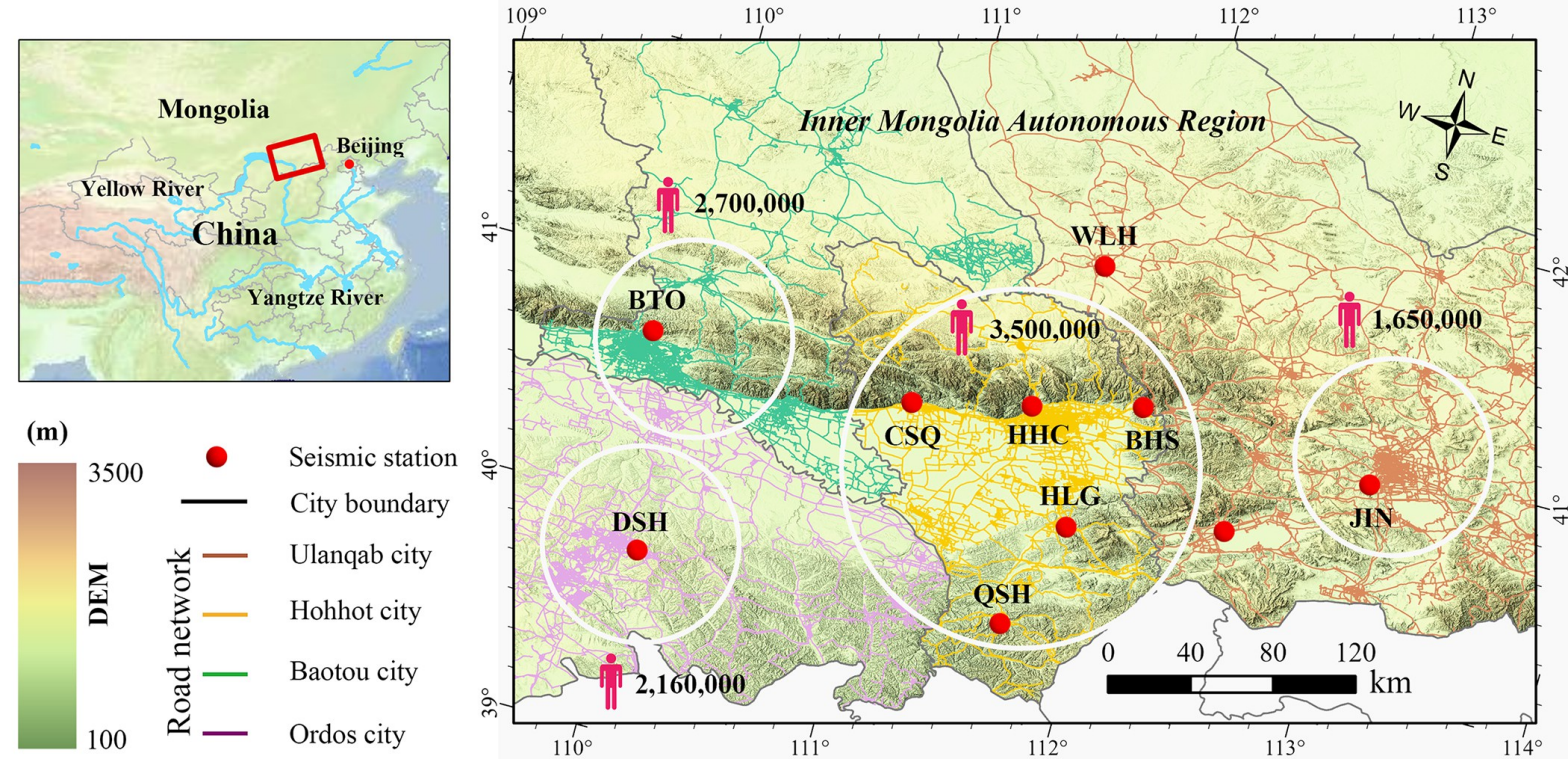
Location of the research region and seismic station, which is in the Northern China, south of the Yinshan mountain, Northeastern part of the Ordos block. The white circle represents the four major cities within the study area (figure was created using the GMT software).

The focal region, as shown in **Fig 1**, is the primary industrialized zone in the Inner Mongolia Autonomous Region of China. This area boasts a well-established transportation system, encompassing both highways and railways, catering to a resident population of roughly 10,000,000 individuals. Moreover, the specific vicinity under scrutiny is marked by a bustling industrial sector, housing numerous prominent noise sources. A Geographic Information System (GIS) has been utilized to generate a visual representation of the infrastructure, depicting the potential distribution of human-induced noise sources in the region. This map illustrates details such as population density, key roadways, and railway networks.

### 2.2 Data source

The Inner Mongolia seismic network includes 48 seismic stations and 1 network center, including 31 broadband recording stations, 15 ultra wideband recording stations, and 2 ultra wideband recording stations. There are 26 ground platforms, 20 cave platforms, and 2 underground platforms; The average distance between stations is 160 km; The stations are equipped with EDAS-24IP, EDAS-24GN, CMG-DM24 and HG-D6 data acquisition; The configured seismometer models include: BBVS-60 seismometers, BBVS-120 seismometers, CTS-1 seismometers, CTS-1EF seismometer, ITC-120A seismometers, JCZ-1T seismometers, and GL-S120B underground seismometer. There are 10 stations in this study area, and the relevant seismic measurement and data acquisition systems are shown in **Table 1**.

**Table 1:** The seismometer and data acquisition system parameters of seismic stations.

Station code	Data acquisition	Seismometer	Range (V)	Conversion factor for data acquisition (uV·counts^-1^)	Seismometer sensitivity (V·s·m^-1^)	Sensitivity of data acquisition (counts·s·m^-1^)
CSQ/QSH/BHS/DSH/WLH	HG-D6	BBVS-60	20	2.384	2000.00	838926175.00
BTO/JIN/LCH	HG-D6	BBVS-120	20	2.384	2000.00	838926175.00
HHC	HG-D6	JCZ-1T	20	2.384	2000.00	838926175.00
HLG	EDAS-24GN	BBVS-60	20	0.149	2000.00	13422818792.00

This study selected ten seismic stations, with six sites belonging to ground platforms and four sites belonging to cave platforms (**Table 2**). The characteristic of these stations is that they are located in the most densely populated areas of Inner Mongolia Autonomous Region, with an average distance of only 20 km between the stations. The stations are distributed in four large cities in Inner Mongolia Autonomous Region, namely Hohhot, Baotou, Ordos, and Ulanqab, with a population of 350,0000, 270,000, 216,00000, and 165,0000 respectively. The total industrial value of these four cities accounts for 59.4% of the total output value of Inner Mongolia Autonomous Region. There is a dense flow of people, road networks, and industrial parks around the site, and environmental noise is objectively present.

**Table 2:** Seismic station information of urban agglomerations in Inner Mongolia.

NO.	Code	Stations	Installation location
1	BTO	Baotou	Cave platform
2	BHS	Baoheshao	Ground platform
3	CSQ	Chasuqi	Ground platform
4	DSH	Dongsheng	Ground platform
5	HLG	Helinger	Cave platform
6	HHC	Hohhot	Cave platform
7	JIN	Jining	Ground platform
8	LCH	Liangcheng	Ground platform
9	QSH	Qingshuihe	Cave platform
10	WLH	Wulanhua	Ground platform

### 2.3 Methods

The approach outlined by McNamara and Buland is widely accepted as the standard for assessing PSDs. To estimate the true variation of noise at a given station, we generate seismic noise PDFs from thousands of PSDs processed [8, 10]. PDF analysis is generated on the basis of PSD analysis. PDF calculates the PSD by averaging and segmenting the observed data. The probability distribution of all PSD curves at each frequency point is represented by color blocks, and all frequency points are collected to generate a PDF. Initially created to monitor seismic station status, this method’s original parameters, along with smoothing and averaging techniques, were designed to minimize storage and computation expenses. However, when applied to scientific research, these features can be restrictive, as demonstrated by recent studies [24].

Focusing solely on the vertical components recorded at the stations, each daily acceleration recording is segmented into 90-minute windows with a 50% overlap. These windows are then subdivided into 15-minute subwindows with a 75% overlap. It has been noted by Anthony et al. that the window length is less critical for higher frequencies and noisier stations, characteristics that are relevant to our current study [24]. A data completeness level above 90% is necessary for each 90-minute window. Transient signals, including earthquakes, are retained in the seismic records as they are infrequent events compared to ambient seismic noise. Anthony et al. demonstrated that while the presence of earthquakes in the recordings can impact the median ambient noise estimates over longer periods (10–50 seconds), there are minimal effects on shorter timeframes [24]. During the preprocessing stage, the data undergo linear detrending, linear gap interpolation, and application of a Hann window to reduce spectral leakage. The computation of the PSD does not involve binning or smoothing. Following the approach of Anthony et al., a one-third-octave averaging is applied to the PSDs [8]. This averaging bandwidth strikes a balance between spectral resolution and the accuracy of broadband noise source characterization within each frequency band. The parameters utilized for evaluating the PSDs in our study can be found in **Table 3**.

**Table 3:** Processing parameters were utilized to assess the PSDs in our research.

Parameter	D’Alessandro et al. [7], McNamara and Buland [10]	Anthony et al. [8]	Fornasari et al. [3]	Present work
Window	60 min	60 min	90 min	60 min
Window overlap	50%	50%	50%	50%
Completeness	-	>90%	>90%	>90%
Subwindow	900 s	819.2 s	900 s	900 s
Subwindow overlap	75%	75%	75%	75%
Detrend	Linear	Linear	Linear	Linear
Gaps	Removed	Zero-pad	Linear interpolation	Linear interpolation
Window type	10% cosine	Hann	Hann	Hann
Binning/smoothing	Yes	None	None	Yes
Average	Overlapped	1/3 octave	1/3 octave	1/3 octave

In order to investigate specific trends in noise levels over time, the PSDs are examined by categorizing them across various time intervals. Analyzing the impact of human-made noise often involves comparing the differences between daytime (08:00–18:00) and nighttime (20:00–07:00 CET; the specified time zone is consistently used in this paper), as well as between weekdays (Monday–Friday) and weekends (Saturday-Sunday). Likewise, the contrasts between summer and winter seasons are evaluated to assess seasonal fluctuations in noise levels. Stations that have data available for both summer and winter periods exceeding 50% are chosen for studying seasonal variations. The relevant statistical parameters are calculated based on the daily median discrepancies within each category.

## 3. Results

Based on the JOPENS system of Inner Mongolia Seismic Network and supported by the waveform data quality analysis software CWQL, ten station parameters are read in real-time from the MySQL database of the JOPENS system, and PSD values and PDF maps of each channel are automatically drawn for each station. Calculating the root mean square (RMS) value of background noise level velocity in the frequency range of 1–20 Hz and store the result; Read the 7-day results data from January 1st, 2023 for analysis.

### 3.1 High noise stations

**Fig 2:**
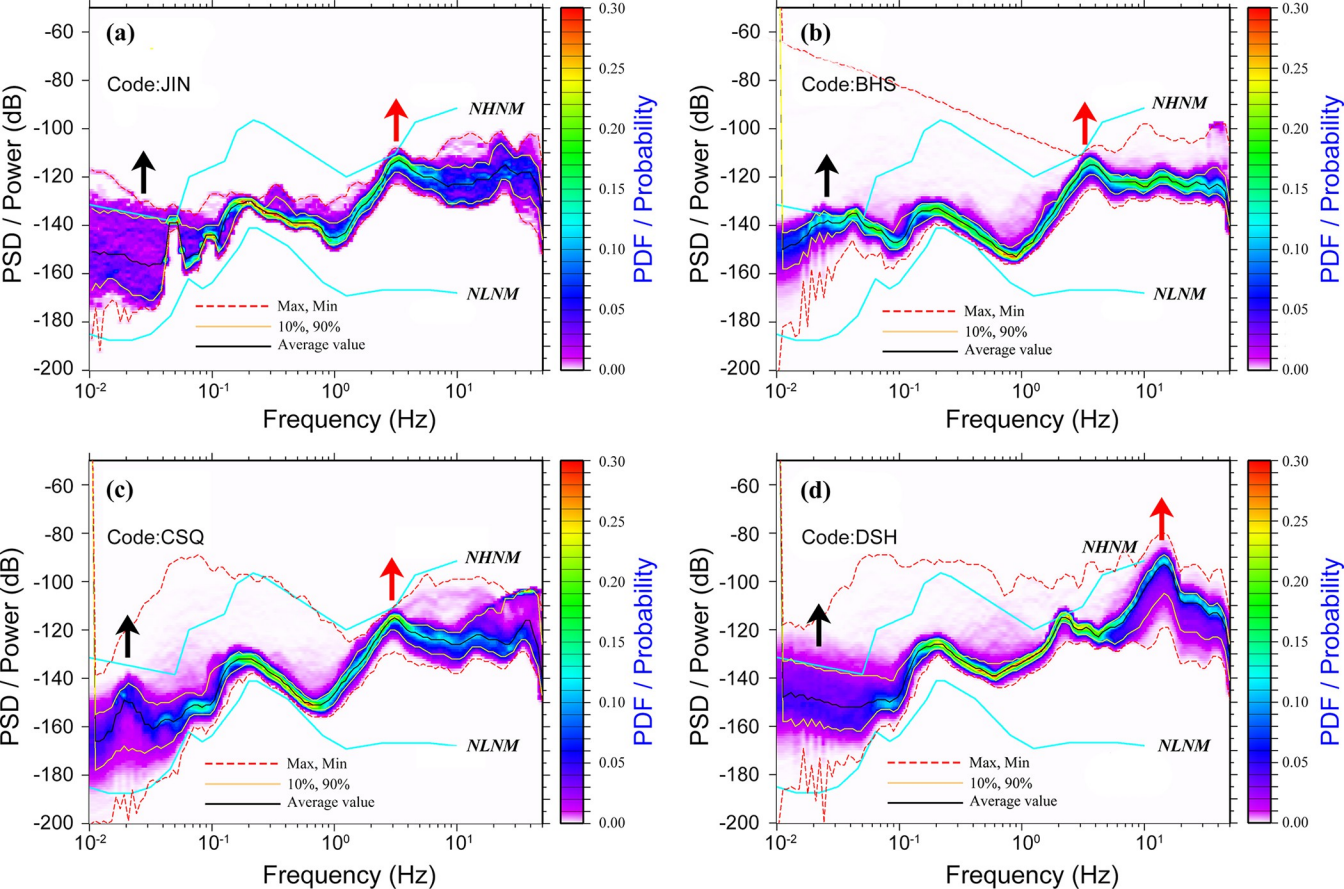
PSD and PDF for the high noise stations, the red arrow represents high-frequency noise, the black arrow represents low-frequency noise, and the arrow pointing upwards indicates a higher noise level, and the arrow pointing downwards indicates a lower noise level (colorbar shows probability values). (a) JIN station, (b) BHS station, (c) CSQ station, and (d) DSH station.

The four high-noise stations have similar PSD shapes and amplitudes in the frequency band above 1 Hz, with a significant step like increase in amplitude, and are close to the global high noise model NHNM (**Fig 2**). Some stations even exceed this model, and the PDF values are relatively concentrated in a certain numerical range. Through on-site investigation, it was found that as the distance between the stations and the interference source increases from far to near, the corner frequency of the PSD value shifts from low frequency to high frequency direction.

High noise stations are all the ground stations, with equipment placed on the surface rather than in deep caves, which makes them susceptible to external noise. The primary sources of external noise are roads, railways, and residential areas (**Table 4**). The main noise source for the JIN station comes from vehicles traveling on the road, located less than 100 m away, with an average maximum noise level of 74 dB. The BHS station’s primary noise sources are vehicle traffic and residential areas, with an average maximum noise level of 68 dB. The CSQ station’s main noise source is residential areas, with an average maximum noise level of 71 dB. The DSH station’s primary noise sources are roads and residential areas, with an average maximum noise level of 81 dB.

**Table 4:** Noise sources around high noise stations (checking time was from 8:00 to 20:00).

Station	Distance from road (m)	Distance from railway (m)	Distance from residential area (m)	Noise value (dB)
JIN	< 100	< 2000	0	58–74
BHS	< 800	/	< 800	41–68
CSQ	< 1900	/	0	42–71
DSH	< 50	/	0	62–81

### 3.2 Low noise stations

**Fig 3:**
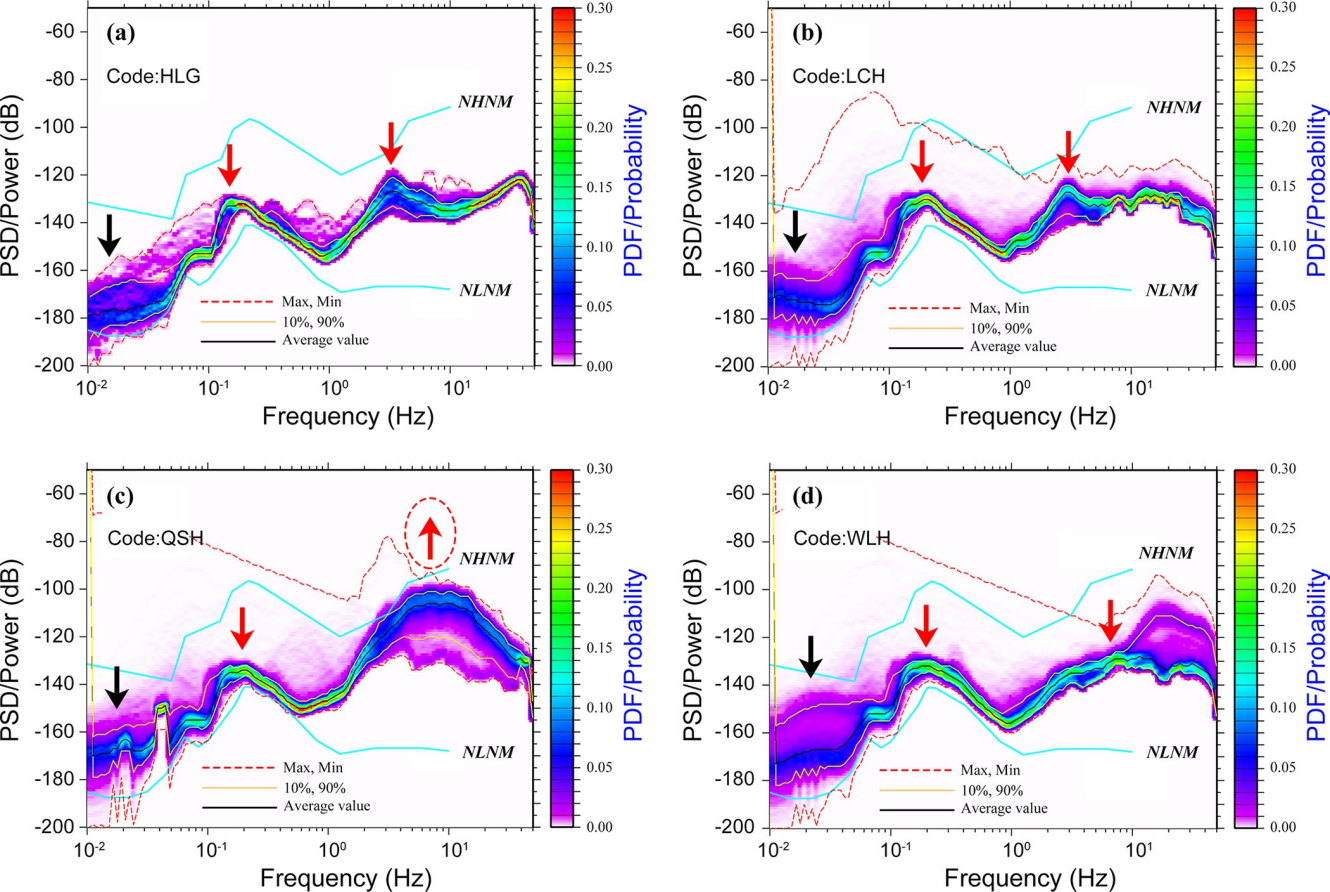
PSD and PDF for the low noise stations, the red arrow represents high-frequency noise, the black arrow represents low-frequency noise, and the arrow pointing upwards indicates a higher noise level, and the arrow pointing downwards indicates a lower noise level (colorbar shows probability values). (a) HLG station, (b) LCH station, (c) QSH station, and (d) WLH station.

The PSD values of low-noise stations are relatively stable throughout the entire frequency range, without the amplitude range of high noise stations (**Fig 3**). The PDF values are more concentrated, except for the >10 Hz range and 0.01–0.1 Hz range of WLH stations, 0.01–0.1 Hz range of LCH stations, as well as the >2 Hz range of QSH stations. This is because WLH and LCH stations are ground observations, and the low-frequency range is affected by temperature, humidity. The >2 Hz range of the QSH station are limited to the influence of road network and industry.

Compared to PDF images of high noise stations, and through field investigation, the low noise stations are far away from villages and county-level or above highways, while most high noise stations are relatively close to villages and county-level or above highways, generally within 0.5 km.

### 3.3 RMS of stations

**Fig 4:**
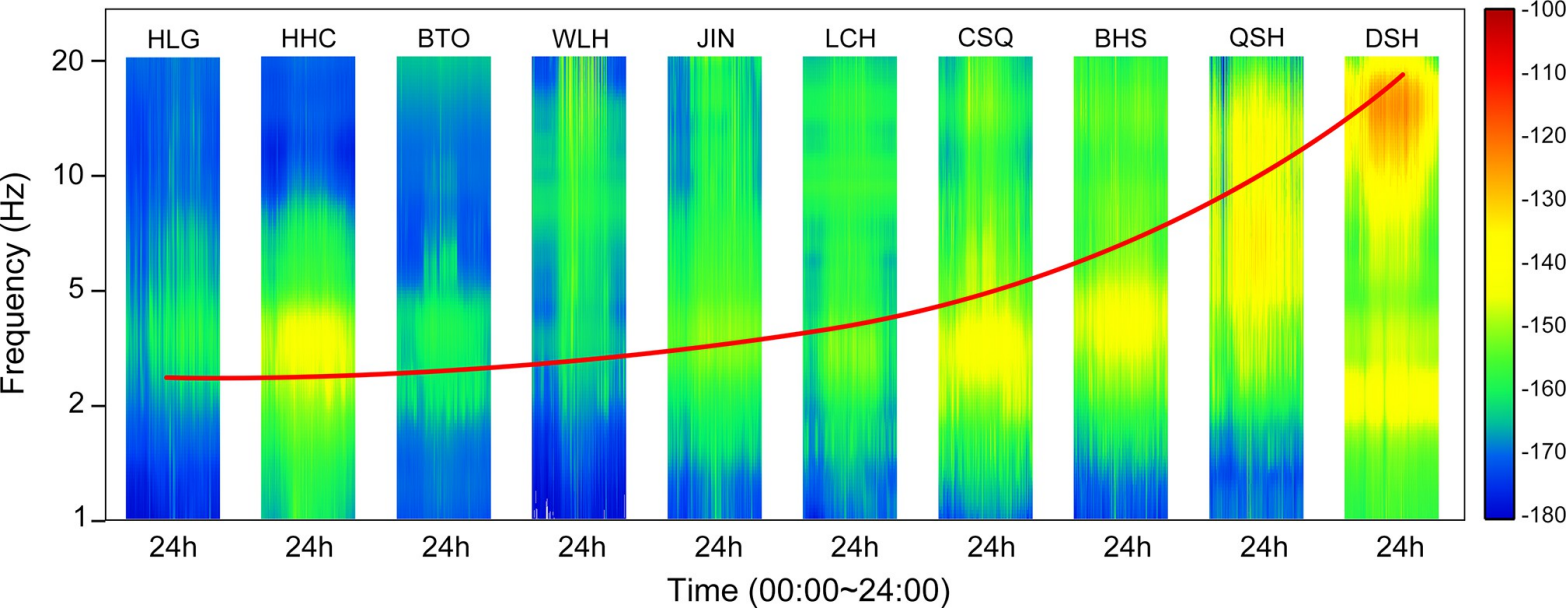
Distribution map of RMS values in the 24h frequency bands of 1–20 Hz for ten stations, and the red curve indicates a gradual increase in the noise frequency of the station (colorbar shows power values(dB)).

In order to quantitatively examine the spatial distribution features of RMS values in the high-frequency band, the RMS values in the 1–20 Hz frequency range were initially computed using the seven-day continuous observation data from ten stations simultaneously at the same time period (**Fig 4**). Among the ten stations in the research area, there are four stations with lower noise levels (HLG, LCH, QSH, WLH) and four stations with higher noise levels (JIN, BHS, CSQ, DSH). Stations with significantly high RMS values are related to the dense population and relatively developed transportation and industry in the region. In areas with sparse population and low industry, the RMS value is significantly lower than other areas, indicating that the noise in the high-frequency range mainly comes from human activities. The difference in RMS values between some stations is more than 50 dB, indicating significant regional differences in RMS values in the high-frequency range.

To further understand the spatiotemporal variation characteristics of RMS values in the region, the 7-day continuous data was divided into nighttime (21:00–8:00) and daytime (8:00–21:00). The spatial distribution of RMS values is basically consistent between day and night, indicating that regional differences in RMS values do not change with day and night. The RMS values of all stations are higher during the day than at night, and there is a significant difference between stations between day and night. This characteristic of day and night difference is consistent with human sleep patterns, indicating that high-frequency background noise is mainly affected by human activities, and the degree of influence varies in different regions.

### 3.4 Source of ambient noise

**Fig 5:**
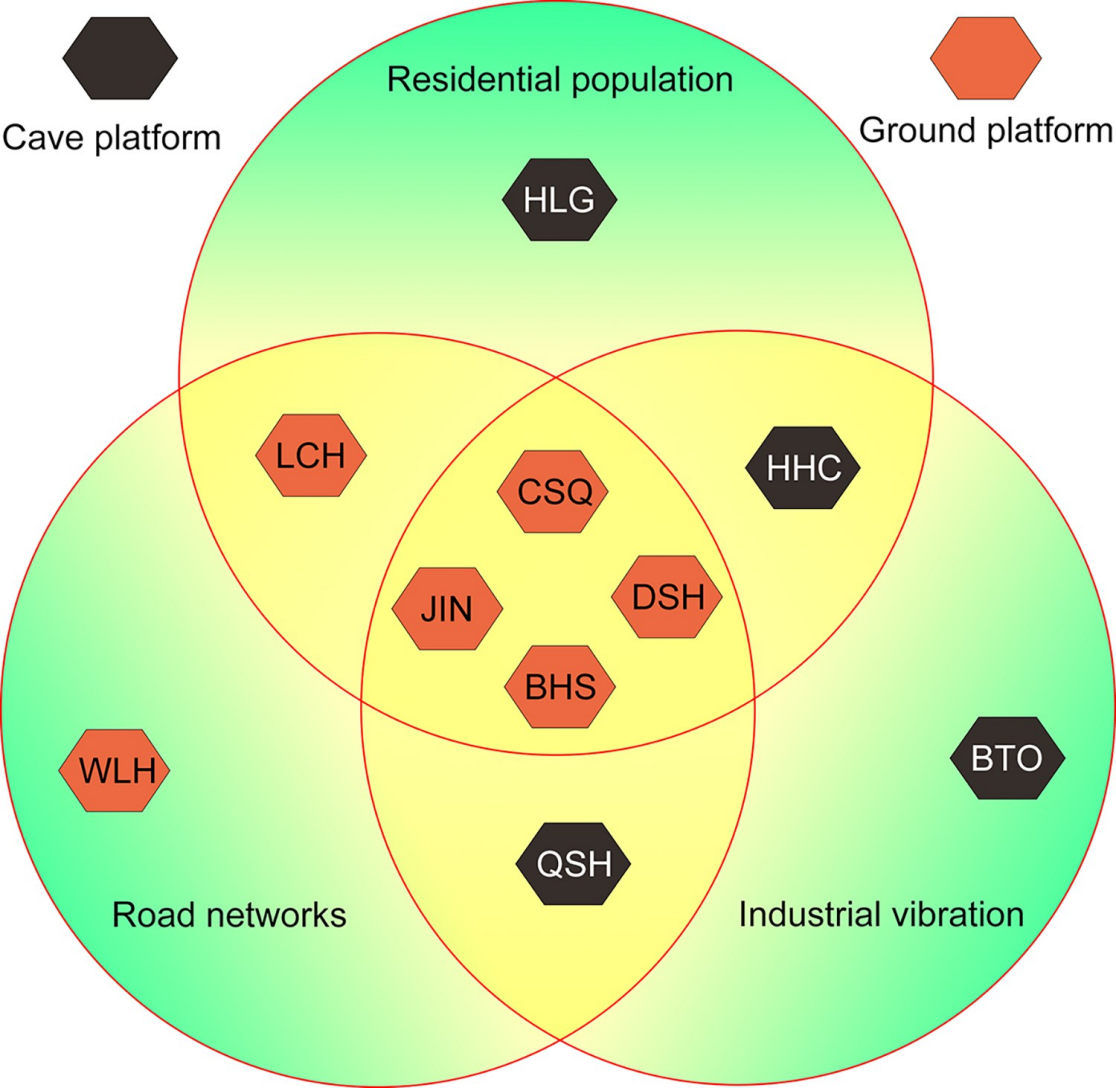
The classification of 1–20 Hz noise sources and the main noise sources of different stations, the closer the stations are to the geometric center point, the greater the influence of the external noise source.

The ambient noise values of the ten stations in the study area vary greatly, and they are all affected by human activities. Within the range of 1–20 Hz, the main sources of ambient noise are human activities, traffic vibrations, and industrial noise. According to on-site investigations, it was found that the distance between the station and the noise source, as well as the intensity of the noise source, are all related to the ambient noise value of the station. Therefore, the types and intensities of noise sources at ten stations were classified (**Fig 5**).

The station on the cave platform has strong anti-interference ability, and the equipment is placed on bedrock with a depth greater than 10 m, filtering out surface noise of general intensity. Therefore, although the actual noise of stations HLG, HHC, and BTO is relatively high, the recorded ambient noise values are relatively low. The station HLG is located in the suburbs of Helingeer County, which being far from the main road or industrial area, with only a small amount of human activity interference, and an ambient noise level of -162 dB. The BTO station is located in the northern part of Baotou City, and the cave is at the foot of Daqing Mountain, which is far from the city center, with less human activity and only the impact of mining noise. The ambient noise level is -158 dB. The station WLH is located in a grassland area with low population density, sparse road network, and less industry, so it is less affected by noise interference, and the ambient noise value is -163 dB.

The station LCH is mainly affected by the combined effects of human activities and traffic vibration. The station HHC is mainly affected by the combined effects of human activities and industrial noise. The QSH of the station is mainly affected by the combined effects of traffic vibration and industrial noise. The four stations (CSQ, BHS, DSH, and JIN) had the highest noise levels, which have been affected by human activities, traffic vibrations, and industrial noise. These stations are located close to densely populated areas, main roads, and industrial area. In addition, these four stations are all ground platforms with weak resistance to external noise, and even have ground amplification effects on external noise.

## 4. Discussion

### 4.1 Frequencies in the range 1–20 Hz

**Fig 6:**
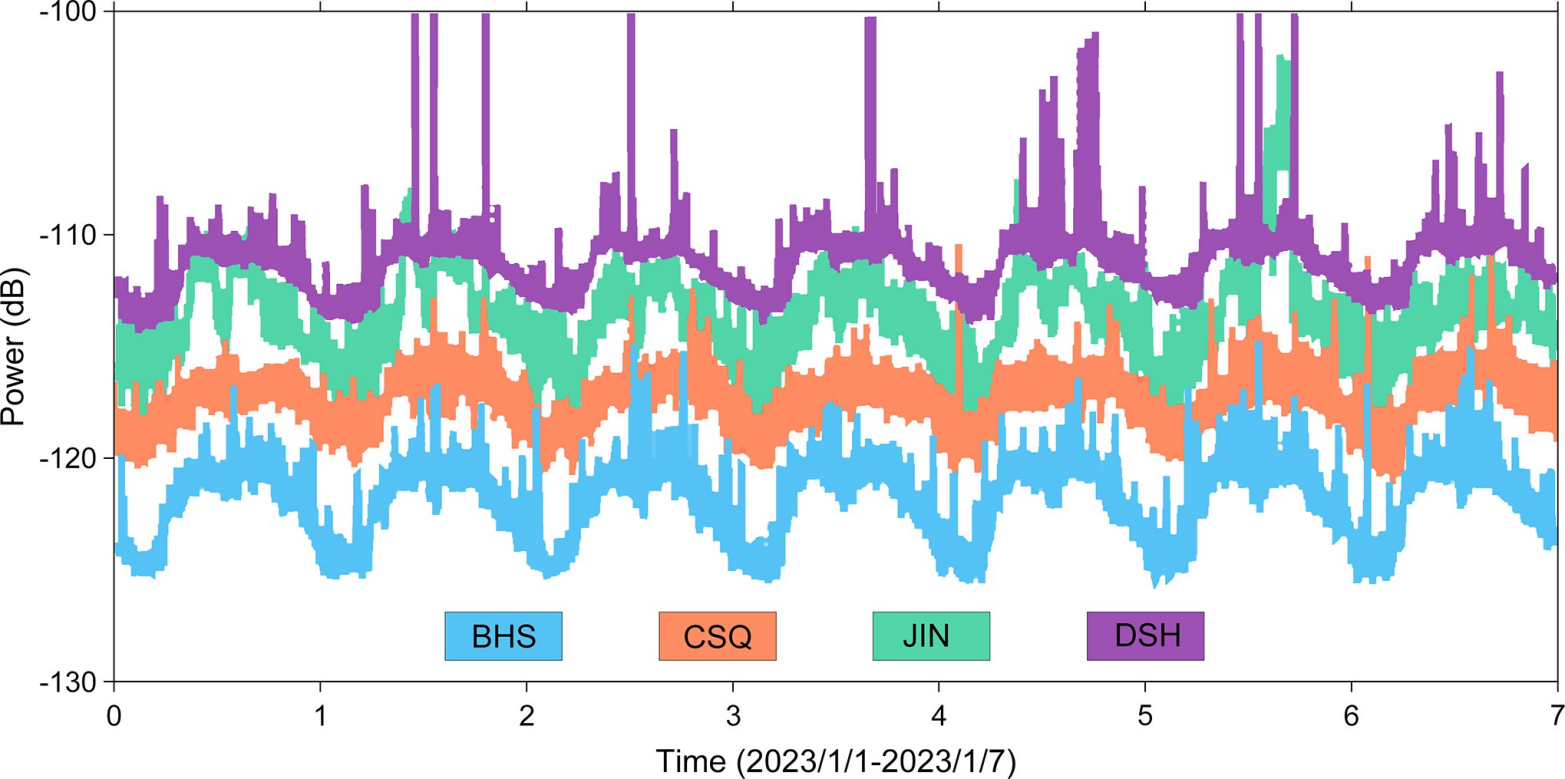
High frequency noise affected by external environment (5.0 Hz), the noise level during the day is significantly higher than that at night.

The seismic background noise originates from various sources. While anthropogenic activities predominantly contribute to high-frequency seismic noise (frequency >1 Hz) [10], weather conditions primarily influence seismic noise at frequencies <1 Hz [25]. Human activities are the primary generators of high-frequency noise, known as cultural noise, resulting from the transmission of energy from traffic and machinery to the Earth. The PDFs illustrating the analyzed stations are presented in **Fig 6**.

Cultural noise diminishes within a few kilometers, causing variations in both the magnitude and characteristics of high-frequency noise at different sites. Stations in close proximity to industrial and infrastructural zones (BHS, CSQ, DSH, JIN, QSH) exhibit the highest noise levels. Specifically, the PDFs of DSH and JIN surpass the New High Noise Model (NHNM) at frequencies between 4–5 Hz. Conversely, stations located in remote settings, such as BTO, HHC, and HLG (they are situated in an underground cavern), as well as WLH in an isolated area and a small village, demonstrate lower noise levels. Station QSH, positioned at an elevation of 1164 m in a cave, exhibits minimal background noise, although disturbances from urban areas are present at frequencies >2 Hz. Additionally, the noise levels at station DSH are influenced by mining activities.

The primary source of vibration for a moving train is the interaction between the rail, wheels, and body. Distinct frequency intervals of 0.8–4 Hz and 8–15 Hz result in the train experiencing lateral movements due to uneven surfaces and vertical motions due to axle hop, respectively. It has been shown that the predominant frequency can shift between 2 Hz and 10 Hz with alterations in the design of the speed reducer and suspension system [22].

### 4.2 Frequencies in the range 0.1–1 Hz

Below 0.1 Hz, the frequency exhibits long-term noise, which is primarily influenced by environmental factors like atmospheric pressure and temperature. Within the 0.1–1 Hz range, the PDFs presented in **Fig 2** are characterized by a prominent peak known as the double-frequency (DF) peak [11]. This peak originates from ocean waves moving in opposite directions with matching periods, resulting in the formation of gravity standing waves with half of the original period. These waves create a non-linear pressure disturbance that travels down to the ocean floor. The level of DF microseisms is contingent upon the magnitude of ocean waves, the extent of the generation area, and the propagation properties [25]. The association between the DF peak and the occurrence of oceanic storms is reinforced by the analysis of the seasonal variations in DF characteristics.

The seasonal noise variability is obvious for frequencies <1 Hz. In winter, noise power exceeds that of summer, with the mode peak shifting towards lower frequencies [26]. The microseismic noise frequency band, ranging from 0.1–1 Hz, has a peak near 0.2 Hz, resulting from wave interactions [27]. Natural sources contribute to the sub-1 Hz band, occasionally overlapping with human-made signals [16]. Seismic free oscillations are mainly caused by oceanic infragravity waves. At the seafloor, atmospheric disturbances create pressure sources, leading to oceanic infragravity waves that can trigger long-period seismic hum (< 0.02 Hz). Wind wave interactions also generate oceanic infragravity waves [28]. Due to depth-dependent behavior and strong coupling with seabed topography, both oceanic and atmospheric influences likely generate microseisms in the study area. A potential link between seismic noise and air pressure, mean wind speed, and wind velocity was investigated. The highest cross-correlation coefficient between seismic noise and wind gusts is 0.62 for the < 0.2 Hz band and 0.59 for the 0.25–1 Hz band [22].

In the central region of Inner Mongolia, the seismic noise characteristics observed in the earthquake stations can be attributed to various natural sources and environmental factors. The long-term noise below 0.1 Hz is primarily influenced by atmospheric pressure and temperature changes, which are common in this area. Additionally, the double-frequency (DF) peak within the 0.1–1 Hz range is a result of ocean waves moving in opposite directions with matching periods, leading to the formation of gravity standing waves that create non-linear pressure disturbances reaching the ocean floor.

The seasonal noise variability is also evident in the central region of Inner Mongolia, particularly for frequencies <1 Hz. During winter months, the noise power exceeds that of summer, and the mode peak shifts towards lower frequencies. This pattern can be linked to the increased wave interactions and stronger atmospheric disturbances occurring during the colder season. Furthermore, the <1 Hz band is affected by both natural sources, such as oceanic infragravity waves, and human-made signals. Wind wave interactions and depth-dependent behavior contribute to the generation of microseisms in this area, further complicating the analysis of seismic noise and its correlation with air pressure, mean wind speed, and wind velocity.

## 5. Conclusions

The ambient noise of ten stations in the urban region of Inner Mongolia was analyzed in this research. The study investigated the features of background seismic noise using the methods of PDF and PSD, in the frequency band of 0.01–20 Hz. Significant fluctuations in noise powers were observed within the 1–20 Hz range, as the recorded noise signals were heavily influenced by human activities.

1. In the 0.01–1 Hz frequency band, cave stations are less affected by temperature and humidity in the section compared to ground stations, and the more deeply the cave is affected, the smaller the impact. The ground platform is greatly affected by temperature and humidity.
2. In the 1–20 Hz frequency band, high noise stations are mainly affected by traffic, industry, and human activities, and their correlation is high. Low noise stations are mainly distributed in remote areas far from villages and transportation, or equipment are installed in deeper caves.
3. Stations located in densely populated areas are greatly affected by ambient noise levels, making it difficult to process data correctly. Human activities, transportation, and industry have all brought significant ambient noise to the stations. In the future, it is possible to consider relocating to remote mountainous areas and using deep buried cave platforms as much as possible to produce high-quality data.
